# Energy expenditure of interruptions to sedentary behavior

**DOI:** 10.1186/1479-5868-8-69

**Published:** 2011-06-27

**Authors:** Ann M Swartz, Leah Squires, Scott J Strath

**Affiliations:** 1 Physical Activity and Health Research Laboratory, Department of Human Movement Sciences, University of Wisconsin-Milwaukee, Enderis Hall Room 453, P.O. Box 413, Milwaukee, WI 53201-0413, USA

## Abstract

**Background:**

Advances in technology, social influences and environmental attributes have resulted in substan-tial portions of the day spent in sedentary pursuits. Sedentary behavior may be a cause of many chronic diseases including obesity, insulin resistance, type 2 diabetes and the metabolic syndrome. Research demonstrated that breaking up sedentary time was beneficially associated with markers of body composition, cardiovascular health and type 2 diabetes. Therefore, the purpose of this study was to quantify the total energy expenditure of three different durations of physical activity within a 30-minute sedentary period and to examine the potential benefits of interrupting sedentary behavior with physical activity for weight control.

**Methods:**

Participants completed four consecutive 30-minute bouts of sedentary behavior (reading, working on the computer, or doing other desk activities) with and without interruptions of walking at a self-selected pace. Bout one contained no walking interruptions. Bout two contained a 1-minute walking period. Bout three contained a 2-minute walking period. Bout four contained a 5-minute walking period. Body composition and resting metabolic rate were assessed.

**Result:**

Twenty males and females (18-39 years) completed this study. Results of the repeated measures analysis of variance with post-hoc testing showed that significantly more energy was expended during each 30 minute sedentary bout with a walking break than in the 30 minute sedentary bout (*p *< 0.05 for all comparisons). On average, participants expended an additional 3.0, 7.4, and 16.5 additional net or activity kilocalories during bouts 2, 3, and 4, respectively compared with bout 1. When extrapolated for a full eight-hour working day, this data shows that an individual would theoretically expend an additional 24, 59 or 132 kilocalories per day, if they stood up and walked at a normal, self selected pace for one, two or five minutes every hour, respectively, compared with sitting for the 8-hour period.

**Conclusions:**

This study demonstrated that making small changes, such as taking a five minute walking break every hour could yield beneficial weight control or weight loss results. Therefore, taking breaks from sedentary time is a potential outlet to prevent obesity and the rise of obesity in developed countries.

## Background

Over the past few decades, technological advances, social influences and environmental attributes have impacted the way we live at home, work and during our leisure time, resulting in substantial portions of the day spent in sedentary pursuits. This shift in lifestyles has impacted individuals of all ages, resulting in significant portions (7.7 hours or 55%) of their day spent in sedentary behavior [1]. Based on well executed epidemiological research, it has been suggested that large amounts of daily sedentary behavior is a challenge to the health of individuals living in industrialized nations [2-6]. More specifically, sedentary behavior may be one of the causes of many modern day chronic diseases including obesity, insulin resistance, type 2 diabetes and the metabolic syndrome [7]. Scientists hypothesize that for those individuals that do not engage in any exercise (39% of population) [8], the risk for these chronic diseases may be increased by simply being more sedentary (sitting, watching TV, etc.) [9].

Animal studies have examined the negative impact of prolonged sitting [10,11]. Hamilton and colleagues have demonstrated that prolonged periods of unloading and lack of muscle contraction resulted in suppression of skeletal muscle lipoprotein lipase activity and reduced glucose uptake in rodents [10,11]. Research with humans has supported this animal work demonstrating that individuals who sit more than 4 hours at a time have a greater risk of diabetes, hypertension, and hyperlipidemia. Furthermore, for those individuals who are physically active, their physical activity did not compensate for the impact that extended sitting had on their health [4,12]. This evidence suggests that lack of muscle contraction and/or energy expenditure may be one of the mediating factors between the published relationship of sedentary behavior and poor metabolic health.

Based on the evidence that prolonged sitting was shown to be detrimental to health, Healy and colleagues [13]examined the association between the number of interruptions (standing up, walking, etc) to sedentary time with markers of metabolic risk. Results demonstrated that independent of total sedentary time, moderate-to vigorous-intensity activity, and mean intensity of activity, more breaks in sedentary time were beneficially associated with indicators of body composition (lower waist circumference, body mass index), and metabolic health (triglycerides and 2-hour post load glucose values). The results of this study highlighted the importance of interrupting prolonged periods of sedentary time and suggested that health may be improved by getting up from your desk at work to walk to the water fountain or break room more times each hour.

The evidence for cross-sectional relationships between sedentary behavior and health is accumulating, and animal studies are providing insight into the mechanisms by which more sedentary time is associated with poor health. However, human studies focusing on the acute effects of breaking up sedentary time are scarce. Therefore, the purpose of this study was to quantify the total energy expenditure of three different durations of physical activity within a 30-minute sedentary period and to examine the potential benefits of interrupting sedentary behavior with physical activity for weight control.

## Methods

### Participants

Twenty males and females between the ages of 18 and 39 years old completed this study. Participants were recruited through posting and distribution of flyers, announcements on the Laboratory website, and from announcements in University classes. Participants were included if they were apparently healthy, not taking any medications that impact metabolism, and had no limitations to walking. Participants were excluded if they had any metabolic, cardiovascular, or pulmonary diseases. All participants read and signed and informed consent document approved by the University Institutional Review Board prior to participation.

### Overview

The design of this experimental study included two Laboratory visits completed within a 14-day period. Participants were asked not to consume any food or calorie containing beverages four hours prior to the visit, not to engage in any exercise 12 hours prior and no vigorous exercise 48 hours prior to the visit, and not ingest any caffeine or take any other stimulants 24 hours prior to the first visit. During the first visit, the informed consent document and a health history and demographics questionnaire were completed as well as anthropometric measures assessed. The participant then completed four consecutive 30-minute bouts of sedentary behavior with and without interruptions of light physical activity, for a total of two hours of monitoring. Sedentary bout one included sitting in a chair at a table for 30 consecutive minutes. Sedentary bout two consisted of 14 minutes of sitting one minute of walking and 15 minutes of sitting, for a total of 30 minutes. Sedentary bout three included 13 minutes of sitting two minutes of walking and 15 minutes of sitting, for a total of 30 minutes. Sedentary bout four consisted of 13 minutes of sitting five minutes of walking and 12 minutes of sitting, for a total of 30 minutes. During the sitting or sedentary periods, the volunteers had the option of reading, working on the computer, or doing other desk activities. During the walking periods, participants walked at their usual (self selected) pace in an interior corridor of a University building while a research assistant followed with a distance wheel and stop watch to calcu-late their average rate of walking.

The second visit took place in the morning after an overnight fast, within 14 days of the first visit. Participants were instructed not to consume any food, calorie containing beverages, caffeine, stimulants, or drugs until the testing was complete and not exercise 12 hours prior to the visit. Body height and mass were again measured and a resting metabolic rate assessment and body composition assessment using dual-energy x-ray absorptiometry were completed.

### Measures

#### Energy Expenditure during Sedentary Bouts

Oxygen consumption, carbon dioxide production and ventilation were measured via indirect calorimetry using the COSMED K4b^2 ^portable metabolic system during each of the four bouts. Use and set up of the system followed manufacturer instructions. Prior to each test and after each battery change, the COSMED K4b^2 ^underwent gas and flow meter calibration according to manufacturer instructions. Participants also wore a polar heart rate monitor to capture heart rate data during the four 30-minute bouts. The COSMED K4b^2 ^has been shown to be a valid measure of energy expenditure as compared to Douglas bags [14].

#### Resting Metabolic Rate

Resting metabolic rate (RMR) was assessed utilizing the flow-through hood technique and analyzed using a ParvoMedics TrueOne metabolic measurement system (Parvomedics, Salt Lake City, UT). Each individual was asked to strictly comply with pre-testing requirements, including no food or beverages for 8 hours prior (excluding water), and no exercise for 12 hours prior. Individuals were assessed while awake after laying supine for a minimum of 30 minutes in a thermo-neutral environment. Minute by minute oxygen consumption, carbon dioxide production and ventilation data were analyzed to determine RMR during a steady state period of 10 minutes or more. Heart rate data was also collected using a standard heart rate chest band and watch receiver. Prior to each test, gas and ventilation calibration of metabolic measurement system were completed based on manufacturer recommendations. The ParvoMedics TrueOne metabolic measurement system has been shown to be a valid measure of oxygen consumption, carbon dioxide production and ventilation [15].

#### Body Composition and Anthropometric Assessments

Body mass and height were measured with minimal clothing and no shoes. Body mass was measured to the nearest 0.01 kg using a physician's balance beam scale (Continental Scale Corporation, Bridgeview, IL) and height was measured to the nearest 0.1 cm using a stadiometer (Continental Scale Corporation, Bridgeview, IL). Body mass index (BMI) was calculated according to the formula body mass (kg) divided by height squared (m^2^). Circumference measurements were taken at the waist (narrowest part of the torso between the most inferior rib and the iliac crest) using a plastic tape fitted with a tension handle. All waist circumference measurements were taken in duplicate to the nearest 0.1 cm at the end of exhalation, with the average measure-ment recorded for analysis.

Three-compartment body composition was assessed via dual energy x-ray absorptiometry (DXA; GE Lunar Prodigy, Madison, WI). DXA has been shown to be a reliable and valid measure of percent body fat, with estimates of body composition within 1 to 3% body fat from multi-component models [16], and coefficient of variation values comparable to those of hydrostatic weighing and air displacement plethysmography [17].

### Data & Statistical Analysis

The Cosmed K4b^2 ^provides breath-by-breath oxygen, carbon dioxide and ventilation data, and calculates VO_2 _and VCO_2_. Data were averaged over 1-minute intervals. Measured RMR was subtracted from measured gross energy expenditure collected during the sedentary bouts to determine net energy expenditure for each bout.

Descriptive statistics were run on all variables. Normality of data was assessed using histograms and tests of skew. Repeated measures analysis of variance was used to compare the total gross energy expenditure, total net energy expenditure, net physical activity energy expenditure and net sitting energy expenditure between the four sedentary bouts. Tukey post-hoc testing was performed to determine significant difference between bouts. Finally, independent T-tests were performed to determine whether group differences were present for VO_2 _values within bouts.

Descriptive statistics are expressed as mean ± standard deviation; all other data are reported as mean ± standard error. Analyses were performed using SPSS^® ^16.0 for Windows (SPSS Inc., Chicago, IL), and the alpha level was set at 0.05.

## Results

### Participant Characteristics

Participants included twenty young men and women (age range 19-38 y) with a BMI ranging from normal to obese and measured body fat percentage ranging from below recommended levels to obese [18,19]. Eighty percent of the participants were Caucasian, 15% were African American, and 5% reported being Pacific Islander. Resting metabolic rates of the participants were at levels that would be expected for young adult males and females (Table 1).

**Table 1:** Descriptive characteristics of participants (N = 20)

Variable	Mean	SD	Minimum	Maximum
Age (y)	28.1	5.7	19.0	38.0
Height (cm)	172.1	9.6	155.0	187.0
Body mass (kg)	82.7	22.2	51.5	136.8
BMI (kg/m^2^)	27.8	6.6	19.8	41.1
WC (cm)	95.5	58.2	66.4	104.9
W:H ratio	0.82	0.17	0.68	0.90
Body fat (%)	24.9	12.7	5.6	46.1
RMR (ml/kg/min)	3.1	0.4	2.6	4.0
RMR (kcal/day)	1759	375	1261	2589

*Note*. BMI, body mass index; WC, waist circumference; W:H ratio, waist to hip ratio; RMR, resting metabolic rate.

### Energy Expended During Sedentary Bouts with and without Activity

On average, participants expended 7.3% or 3.2 additional gross kilocalories during bout 2, 17% or 7.6 additional gross kilocalories during bout 3, and 37% or 16.6 additional gross kilocalories during bout 4 compared with bout 1 (Table 2). When taking into consideration resting energy expenditure, participants expended 40% or 3.0 additional net kilocalories during bout 2, 99% or 7.4 additional net kilocalories during bout 3, and 220% or 16.5 additional net kilocalories during bout 4 compared with bout 1. Finally, when expressing the net energy expenditure relative to kilograms of lean body mass, participants expended 43%, 86%, and 200% more net kilocalories per kg lean body mass during bouts 2, 3, and 4, respectively, than during bout 1.

**Table 2:** Energy expenditure during sedentary bouts with and without an activity break (mean ± SE, N = 20).

	Bout 1	Bout 2	Bout 3	Bout 4
Gross EE (kcal/30 min)	43.9 ± 2.09^b, c, d^	47.13 ± 2.2^a, c, d^	51.49 ± 3.02^a, b, d^	60.45 ± 3.51 ^a, b, c^
Net EE (kcal/30 min)	7.49 ± 1.23 ^b, c, d^	10.49 ± 1.38 ^a, c, d^	14.85 ± 1.61 ^a, b, d^	23.96 ± 2.04 ^a, b, c^
Relative Net EE (kcal/30 min/kg lean body mass)	0.14 ± 0.03 ^b, c, d^	0.20 ± 0.03 ^a, c, d^	0.26 ± 0.03 ^a, b, d^	0.42 ± 0.05^a, b, c^

^a ^significantly different than bout 1 (*p *< .05).

^b ^significantly different than bout 2 (*p *< .05).

^c ^significantly different than bout 3 (*p *< .05).

^d ^significantly different than bout 4 (*p *< .05).

*Note*. Bout 1 consisted of 30 consecutive minutes of sitting. Bout 2 consisted of 14 minutes of sitting, 1 minute of walking, and 15 minutes of sitting. Bout 3 consisted of 13 minutes of sitting, 2 minutes of walking, and 15 minutes of sitting. Bout 4 consisted of 13 minutes of sitting, 5 minutes of walking, and 12 minutes of sitting.

Further examination and extrapolation of the net energy expenditure from breaking up sedentary time suggests the potential for significant caloric expenditure over time, assuming weight, physical activity and diet stability (Table 3). When extrapolated for a full eight-hour working day, if an individual stood up and walked for 1-minute every hour, they would theoretically expend an additional 24 kilocalories per day, or an additional 120 kilocalories per week compared with sitting for 8 hours. Further, standing up and walking at a normal, self selected pace for two to five minutes once every hour during an eight hour work day would theoretically result in an additional 59 to 132 kilocalories, respectively expended during an eight hour work day, or 296 to 660 additional kilocalories per week, compared with sitting.

**Table 3:** Energy expenditure extrapolations for disrupting sedentary time with walking breaks.

Time period	1 minute of walking/30 minutes	2 minutes of walking/30 minutes	5 minutes of walking/30 minutes
1 hour (kcals)	6.0	7.4	16.5
8 hour workday (kcals)	24	59.2	132
1 week of 8 hour workdays (kcals)	120	296	660
1 month of 8 hour workdays (kcals)	480	1184	2640
6 months of 8 hour workdays (kcals)	2880	7104	15840
12 months of 8 hour workdays (kcals)	5760	14208	31680

Sitting accounted for 100% of the net energy expenditure in bout 1, 83% of the net energy ex-penditure in bout 2, 65% of the net energy expenditure in bout 3, and 38% of the net energy expenditure in bout 4. Sitting VO_2 _values during bouts 2 (*p *= .01) and 3 (*p *= .026) for all participants, bout 2 for females (*p *= .005) and bout 3 for males (*p *= .028) were significantly higher than bout 1. Further, bout 3 was significantly higher than bout 2 for males only (*p *= .014).

The walking break accounted for 17%, 32%, and 62% of the net VO_2 _in bouts 2, 3 and 4 (Table 4). Total net VO_2 _and net physical activity VO_2 _values were significantly higher from bout 1 to bout 2 to bout 3 to bout 4 for all participants, female participants and male participants. While net physical activity VO_2 _continually increased as the walking duration increased, the increase in energy expenditure was not a proportional increase in time spent walking. During bout 3, time spent walking doubled (From 1 minute to 2 minutes), however net physical activity VO_2 _increased by 154%. Additionally, during bout 4, participants spent 5 minutes walking, compared with the 2 minutes spent walking in bout 3 and net physical activity VO_2 _increased by 217%. There were no significant differences between genders for total net VO_2_, net sitting VO_2 _or net physical activity VO_2 _for any of the bouts.

**Table 4:** Oxygen consumption of walking and sitting behaviors during sitting bouts with and without a walking break (mean ± SE).

	Bout 1	Bout 2	Bout 3	Bout 4
Total Gross O_2 _consumption	ml/kg/30 min	ml/kg/30 min	ml/kg/30 min	ml/kg/30 min
All	113.4 ± 4.0 ^b, c, d^	122.4 ± 3. 9 ^a, c, d^	132.9 ± 3.3 ^a, b, d^	156.2 ± 3.8 ^a, b, c^
Females	112.2 ± 5.3 ^b, c, d^	124.7 ± 5.8 ^a, d^	130.8 ± 5.0 ^a, d^	155.9 ± 5.9 ^a, b, c^
Males	114.5 ± 6.1 ^b, c, d^	120.1 ± 5.2 ^a, c, d^	135.0 ± 4.5 ^a, b, d^	156.4 ± 4.7 ^a, b, c^
Total Net O_2 _consumption	ml/kg/30 min	ml/kg/30 min	ml/kg/30 min	ml/kg/30 min
All	19. 8 ± 3.4 ^b, c, d^	28.8 ± 3.3 ^a, c, d^	39.3 ± 2.9 ^a, b, d^	62.6 ± 2.9 ^a, b, c^
Females	22.5 ± 4.9 ^b, c, d^	35.0 ± 4.6 ^a, c, d^	41.1 ± 4.0 ^a, b, d^	66.2 ± 4.1 ^a, b, c^
Males	17.0 ± 4.9 ^b, c, d^	22.6 ± 4.6 ^a, c, d^	37.5 ± 4.0 ^a, b, d^	58.9 ± 4.1 ^a, b, c^
Net Sitting O_2 _consumption	ml/kg/30 min	ml/kg/29 min	ml/kg/28 min	ml/kg/25 min
All	19. 8 ± 3.4 ^b, c^	23.8 ± 3.1^a^	25.4 ± 2.5^a^	23.9 ± 2.0
Females	22.5 ± 4.9 ^b^	29.7 ± 4.4^a^	25.7 ± 3.6	26.8 ± 2.6
Males	17.0 ± 4.9 ^c^	17.9 ± 4.4	25.1 ± 3.6^a, b^	21.0 ± 6.7
Net PA O_2 _consumption		ml/kg/min	ml/kg/2 min	ml/kg/5 min
All	--	5.0 ± 0.3 ^c, d^	12.7 ± 0.4 ^b, d^	38.7 ± 1.5 ^b, c^
Females	--	5.4 ± 0.4 ^c, d^	13.0 ± 0.6 ^b, d^	39. 4 ± 2.1 ^b, c^
Males	--	4.6 ± 0.4 ^c, d^	12.4 ± 0.6 ^b, d^	38.0 ± 2.1 ^b, c^

^a ^significantly different than bout 1 (p < .05).

^b ^significantly different tan bout 2 (p < .05).

^c ^significantly different than bout 3 (p < .05).

^d ^significantly different than bout 4 (p < .05).

*Note*. Bout 1 consisted of 30 consecutive minutes of sitting. Bout 2 consisted of 14 minutes of sitting, 1 minute of walking, and 15 minutes of sitting. Bout 3 consisted of 13 minutes of sitting, 2 minutes of walking, and 15 minutes of sitting. Bout 4 consisted of 13 minutes of sitting, 5 minutes of walking, and 12 minutes of sitting.

**Figure 1:**
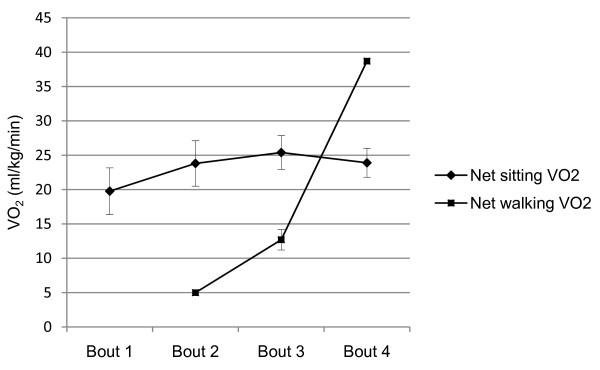
**Net sitting and walking VO_2 _by bout for all participants (N = 20)**. ^a ^significantly different than net walking VO_2 _(p < .001). ^b ^significantly different than net sitting VO_2 _(p < .001). *Note*. Bout 1 was 30 consecutive minutes of sitting. Bout 2 was 14 minutes of sitting, 1 minute of walking, and 15 minutes of sitting. Bout 3 was 13 minutes of sitting, 2 minutes of walking, and 15 minutes of sitting. Bout 4 was 13 minutes of sitting, 5 minutes of walking, and 12 minutes of sitting.

Figure 1 shows that significantly higher oxygen consumption occurred during the 29 and 28-minute sitting portion of bouts 2 and 3, respectively compared with the 1 and 2-minute walking portions of those bouts (both *p *< .001). Significantly higher oxygen consumption occurred during the five-minute walking bout compared with the 25 minutes of sitting in bout 4 (*p *< .001).

### Speed of walking During Activity Breaks

**Figure 2:**
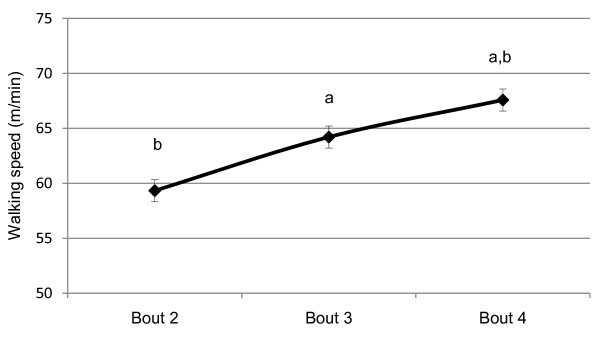
**Self selected walking speed during breaks in sedentary bouts (mean ± SE)**. ^a ^significantly different than bout 2 (p < .05). ^b ^significantly different than bout 3 (p < .05). *Note*. Bout 1 consisted of 30 consecutive minutes of sitting. Bout 2 consisted of 14 minutes of sitting, 1 minute of walking, and 15 minutes of sitting. Bout 3 consisted of 13 minutes of sitting, 2 minutes of walking, and 15 minutes of sitting. Bout 4 consisted of 13 minutes of sitting, 5 minutes of walking, and 12 minutes of sitting.

The mean walking speed during each walking break gradually increased as the walking bout duration increased (Figure 2). During the 1-minute walking bout (bout 2), participants walked an average of 59.3 ± 1.5 m/min. During the 2-minute walking break (bout 3), participants walked significantly faster than during the 1-minute walking break (bout 2; 64.2 ± 1.8 m/min; *p *< .001). Finally, during the 5-minute walking break (bout 4), participants walked significantly faster than during the 2-minute walking break (bout 3; 67.6 ± 1.9 m/min; *p *= .001).

## Discussion

Recent research has focused on the detrimental effects of a sedentary lifestyle. Researchers have hypothesized that breaking up sedentary bouts of behavior with physical activity will improve the health of sedentary individuals. However, the mechanisms that result in health improvements have not been elucidated. Therefore, the primary aim of this study quantify the total energy expenditure of three different durations of physical activity within a 30-minute sedentary period and to examine the potential benefits of interrupting sedentary behavior with physical activity for weight control. Our results demonstrate that standing up and walking at a usual, self-selected pace for one minute during a 30-minute sitting period resulted in an additional 3.0 kilo-calorie net expenditure compared with 30 minutes of sitting. When extrapolated for a full 5 day, eight-hour per day working week, if an individual stood up and walked for 1-minute every hour, they would theoretically expend an additional 120 kilocalories per week compared with sitting for 8 hours. Further, standing up and walking at a normal, self selected pace for two and five minutes once every hour during an eight hour work day would result in 296 and 660 additional kilocalories per week, compared with sitting. This level of energy expenditure is likely to have an important impact on weight maintenance, or even possibly weight loss.

These results have tremendous public health relevance. In 2003, Hill et al suggested that most of the population weight gain that we have seen over the past few decades could be eliminated by some combination of increasing energy expenditure and reducing energy intake by 100 kilocalories per day [20]. Based on the data in this study, if individuals who have sedentary occupations, stood up and walked for at least 5 minutes every hour (walk to the water fountain, to a colleagues desk, etc.), they would attain this theoretical threshold to prevent weight gain and potentially positively impact chronic disease.

Data from this study showed that if an individual took a 1-minute break from sitting every half hour, they would theoretically expend 48 more kilocalories compared with sitting, and an individual who stood up and walked for 2 minutes every hour would expend an additional 59 kilocalories per day, a caloric difference of 11 kilocalories, with no difference in time. Therefore, our data do not lend direct support to the results of Healy et al. [13] who demonstrated that, independent of total time spent being sedentary and time spent in moderate-to vigorous-physical activity, more breaks in sedentary time were associated with a more beneficial waist circumfe-rence, body mass index, triglyceride level and 2-h post load glucose level. Our data, showing an extrapolated difference of 11 kilocalories per day expenditure between a 1-minute break every half hour and a 2-minute break every hour suggest that the more beneficial metabolic profile associated with more frequent breaks may not be due to differences in energy expenditure. However, it should be noted that breaks in the Healy et al study were at least 1-minute in length with an average duration of 4.5 minutes, were light intensity, and were frequent (average of 86 breaks during the day) [13]. Together these data suggest that muscle contractions involved in standing up and sitting down may significantly contribute to the beneficial associations seen with more frequent breaks by Healy et al [13].

During the walking breaks in bouts 2, 3 and 4 average oxygen consumption of all participants was 5.0 ml/kg/min, 12.7 ml/kg/min and 38.7 ml/kg/min. The average walking speeds of all participants were 2.2 mph, 2.4 mph, and 2.5 mph. The compendium of physical activities [21] documented "walking when gathering things at work ready to leave" as 3.0 METS or 10.5 ml/kg/min, "walking less than 2.0 mph on a firm surface" as 2.0 METS or 7.0 ml/kg/min, and "walking 2.5 mph on a flat surface" as 3.0 METS or 10.5 ml/kg/min. Although our calculations of ml/kg/min from compendium data assumed a resting metabolic rate of 3.5 ml/kg/min while measured resting metabolic rate for this study was 3.1 ml/kg/min, our walking results are comparable to the compendium of physical activities.

There are a few important strengths and limitations to take into account while considering the results of this study. First, the primary dependent variable, net energy expenditure, was assessed using indirect calorimetry and calculated using measured resting metabolic rate. Second, although the study population was small, the observed power was calculated at over 0.8 for all repeated measures performed in this study. Third, the focus of the current study was focused on disrupting sedentary behavior within a regular working day. Current results are delimited to those 20-39 years of age. However, it is likely that the increase in measured energy expenditure associated with disrupting sedentary behavior will carry over to individuals over the age of 39 years. Finally, although data was collected in a lab-setting, this intervention allowed the participants to choose a sedentary desk activity and walk at their own pace during breaks, to better mimic the daily activities of an individual with a sedentary job.

These data do not address a number of very important considerations that should be taken into account in future investigations. First, during this intervention, participants were asked to interrupt their sedentary time with walking behavior. In real world situations, this may or may not happen. For instance, an individual may choose to break up their sedentary time by standing to talk with a colleague or walking to the break room to get a snack. In these situations, the energy expenditure would most likely be less, and if the individual consumed calories during this time, i.e. with a snack, the net consumption may well outweigh the net expenditure. Understanding more about how individuals will choose to break up sedentary time will, in addition to the results of this paper, provide good insight into designing interventions to break up sedentary time in order to positively impact health.

## Conclusions

Emerging technologies and customs within the past 50 years that promote sedentary behavior such as TV remotes, drive-thru windows, and computers have contributed to an increase in sitting time and also perhaps to the rise in obesity levels in developed countries. Tactics to compensate for a sedentary lifestyle and therefore facilitate weight-gain prevention need to be promoted. This study demonstrated that for an individual who worked 50 weeks a year, 5 days a week, 8 hours a day and has a desk job, standing up and walking five mi-nutes per hour during every work day, would equate to approximately 33,000 additional kilocalories expended per year. Assuming no changes in diet, other physical activity or metabolism, this could result in 9.4 lbs of body weight (assuming 3500 kcals/pound). Through making small changes like this, an individual could yield beneficial weight control or weight loss results. Therefore, taking breaks from sedentary time is a potential outlet to prevent obesity and the rise of obesity in developed countries.

## Competing interests

The authors do not have any financial or non-financial competing interests.

## Authors' contributions

AMS contributed significantly to the conception and design of the study, analysis and interpreta-tion of data, and drafting of the manuscript. LS contributed significantly to the acquisition of data, analysis and interpretation of data, and critical revision of the manuscript. SJS contributed significantly to the conception and design of the study, analysis and interpretation of data, and critical revision of the manuscript. All authors have given final approval for publication.
